# Preemptive anticoagulation during antenatal pulmonary embolism diagnostics in a community setting: retrospective cohort study

**DOI:** 10.1016/j.rpth.2025.102695

**Published:** 2025-01-31

**Authors:** Aidan R. Campbell, Cole J. Florio, Grace V. Heringer, Sara T. Woldemariam, Scott D. Casey, William B. Stubblefield, Lauren M. Westafer, Edward Qiao, Cydney E. Middleton, Lara Zekar, Nachiketa Gupta, Madeline J. Somers, Mary E. Reed, Nareg H. Roubinian, Ashok P. Pai, Jeffrey D. Sperling, David R. Vinson

**Affiliations:** 1 Kaiser Permanente CREST Network, Pleasanton, California, USA; 2 Department of Microbiology and Molecular Genetics, University of California, Davis, California, USA; 3 Department of Neurobiology, Physiology and Behavior, University of California, Davis, California, USA; 4 Department of Obstetrics and Gynecology, Kaiser Permanente Oakland Medical Center, Oakland, California, USA; 5 Kaiser Permanente Northern California Division of Research, Pleasanton, California, USA; 6 Department of Emergency Medicine, Kaiser Permanente Vallejo Medical Center, Vallejo, California, USA; 7 The Permanente Medical Group, Pleasanton, California, USA; 8 Department of Emergency Medicine, Vanderbilt University Medical Center, Nashville, Tennessee, USA; 9 Department of Emergency Medicine and Department of Healthcare Delivery and Population Science, University of Massachusetts Chan Medical School-Baystate, Springfield, Massachusetts, USA; 10 California Northstate University College of Medicine, Elk Grove, California, USA; 11 Department of Emergency Medicine, UC Davis Health, Sacramento, California, USA; 12 Department of Emergency Medicine, Kaiser Permanente Redwood City Medical Center, Redwood City, California, USA; 13 Department of Pulmonary and Critical Care Medicine, Kaiser Permanente Oakland Medical Center, Oakland, California, USA; 14 Department of Hematology and Oncology, Kaiser Permanente Oakland Medical Center, Oakland, California, USA; 15 Department of Maternal and Fetal Medicine, Kaiser Permanente Modesto Medical Center, Modesto, California, USA; 16 Department of Emergency Medicine, Kaiser Permanente Roseville Medical Center, Roseville, California, USA

**Keywords:** anticoagulants, computed tomography angiography, perfusion imaging, pregnancy, pulmonary embolism

## Abstract

**Background:**

Society recommendations for preemptive (or empiric) anticoagulation during antenatal pulmonary embolism (PE) diagnostics rely on expert opinion, which varies widely across guidelines. The American College of Chest Physicians (CHEST), for example, recommends preemptive anticoagulation when PE is highly suspected or when a delay in imaging is anticipated. The American College of Obstetricians and Gynecologists, however, makes no mention of preemptive anticoagulation for suspected PE in their practice bulletin on thromboembolism in pregnancy. Patterns of preemptive anticoagulation in pregnancy are unknown.

**Objectives:**

To describe the prevalence of and CHEST-based eligibility for preemptive anticoagulation in pregnancy.

**Methods:**

This retrospective cohort study was undertaken across 21 United States community hospitals from October 1, 2021 through March 30, 2023. We included pregnant adults without COVID-19 undergoing definitive diagnostic PE imaging. We used pregnancy-adapted Geneva scores to calculate pretest probability as a proxy for suspicion.

**Results:**

We included 326 patients: median age, 31.0 years; 51% were in the third trimester. Diagnostic settings included emergency departments (*n* = 254; 78%), Labor & Delivery (*n* = 65; 20%), and outpatient clinics (*n* = 7; 2%). Median time from emergency department computed tomography order to results was 1.40 hours (IQR: 0.78, 2.06). Prevalence of confirmed or presumed PE was low (*n* = 8; 2.5%). Only 2 patients (0.6%) received preemptive anticoagulation, whereas by CHEST criteria, 34 patients (10.4%) were eligible.

**Conclusion:**

We found rare use of preemptive anticoagulation during antenatal PE diagnostics in this imaged cohort with low PE prevalence and rapid access to diagnostic imaging. More research is needed to explore setting-specific variation in preemptive anticoagulation use.

## Essentials

• Preemptive anticoagulation (AC) is advised for some with suspected pulmonary embolism (PE).
• We describe the prevalence of preemptive AC among pregnant patients imaged for PE.
• Only 2 of 326 pregnant patients (<1%) were given preemptive AC when 34 were eligible.
• These results should spur an evaluation of preemptive AC in one’s own practice setting.

## 1 Introduction

Pulmonary embolism (PE) occurs in approximately 1 in every 3000 pregnancies and is a leading cause of maternal mortality in the United States (U.S.) and the United Kingdom [[1], [2], [3], [4], [5]]. Anticoagulation is critical to optimizing outcomes in the treatment of acute PE during pregnancy [[6], [7], [8]]. To avoid treatment delays, many professional societies recommend starting preemptive (or empiric) anticoagulation while awaiting diagnostic results [[9], [10], [11], [12], [13], [14], [15], [16], [17]]. In the absence of direct evidence, societies have relied on expert opinion. For example, the American College of Chest Physicians (CHEST) made a weak recommendation for selective use of preemptive anticoagulation based on low-quality evidence (Grade 2C) in their 2012 Antithrombotic Therapy and Prevention of Thrombosis, 9th edition [15]. Without new evidence over the ensuing years, their preemptive anticoagulation recommendations were not revised for the first (2016) or second (2020) updates [18,19].

Lack of evidence may explain the diversity of guideline recommendations, written by experts with differing opinions [9,10,[12], [13], [14], [15], [16], [17]]. The American College of Obstetricians and Gynecologists (ACOG) and the American Society of Hematology do not discuss preemptive anticoagulation during antenatal PE diagnostics [12,20]. Several recent reviews of PE in pregnancy are likewise silent on the issue [[6], [7], [8]].

An evidence deficit and diversity of expert opinion are not the only factors weakening the case for antenatal preemptive anticoagulation. The low prevalence of PE among pregnant patients undergoing diagnostic imaging in the U.S. argues against widespread anticoagulation before imaging results are known [21]. One suggested indication for preemptive anticoagulation is when imaging is “not available on a timely basis” [16]. This delay factor becomes less relevant as more facilities have around-the-clock access to computed tomography pulmonary angiography (CTPA).

These factors raise questions about the suitability of antenatal preemptive anticoagulation to the emergency department (ED) and Labor and Delivery (L&D) unit of medical centers with around-the-clock diagnostic imaging. We sought to evaluate the prevalence of preemptive anticoagulation in pregnancy, a phenomenon not previously well described, particularly in U.S. community medical centers with low prevalence of PE and timely access to CTPA. We also sought to estimate the percentage of patients who would have met the 2012 CHEST criteria for preemptive anticoagulation. Results of this study may inform a reassessment of contemporary practice patterns and the applicability of society guidelines.

## 2 Methods

### 2.1 Design and setting

We conducted a retrospective cohort study called APED (Antenatal PE Diagnostics) [22,23] across 21 Kaiser Permanente (KP) Northern California community medical centers and associated clinics, which collectively care for over 4.5 million health plan members. Members have demographic and socioeconomic characteristics similar to local and state populations [24]. Diagnostic decisions were at the discretion of treating clinicians. The KP Northern California Institutional Review Board approved the study and waived the requirement for informed consent.

The large majority of pulmonary vascular imaging was ordered by emergency medicine physicians working in the ED and obstetricians working in L&D. These hospital-based clinicians had 24-hour access to routine laboratory tests (including D-dimer), plain films, CTPA, and specialty consultation [25]. Formal compression ultrasonography (CUS) from hospital-based radiology departments was available daily from 0700 to 2100, and lung scintigraphy (ie, ventilation/perfusion [V/Q] scans) from nuclear medicine from 0800 to 1700. Off-hours availability varied by hospital and often required on-call specialty approval. Clinicians in outlying outpatient clinics could order any PE diagnostic test, including pulmonary vascular imaging [26]. Onsite access, however, could be limited, and varied by clinic, sometimes requiring patients to wait a day or more and/or to travel to a nearby facility to complete their diagnostic evaluation.

### 2.2 Study population

The larger APED study population consisted of ambulatory pregnant health plan members ≥18 years of age who underwent 1 of 4 PE diagnostic tests (D-dimer, CUS, CTPA, or lung scintigraphy) from October 1, 2021, through March 30, 2023, with at least 1 of the following PE-suggestive symptoms [22]: new or worsening dyspnea, chest or thoracic pain, hemoptysis, palpitations, syncope, or well-defined presyncope [27]. We identified pregnant persons using the KP Northern California Division of Research Perinatal Research Unit’s Perinatal Obstetric Database. We used KP’s clinical databases to identify the subset undergoing PE testing. We used validated algorithms to help identify patients undergoing pulmonary vascular imaging for suspected PE [28]. We manually reviewed the electronic health records of test recipients to determine APED eligibility. We excluded patients not known to be pregnant at the time of testing, with recent or impending pregnancy loss (as diagnosticians approach these patients differently than patients with a living fetus), already receiving therapeutic anticoagulation, or who left before completing the agreed diagnostic evaluation. We excluded pregnant patients who did not commence their PE diagnostic evaluation until after hospitalization, as APED focused on ambulatory, not hospitalized, patients. Additionally, we excluded patients with COVID-19 from the primary APED analyses because this infection alters conventional PE diagnostics.

This current analysis was limited to APED participants who underwent definitive diagnostic imaging tests for PE sufficient to rule in venous thromboembolism (VTE) (via positive CUS or pulmonary vascular imaging) or rule out PE (via negative pulmonary vascular imaging). We did not include patients who received D-dimer testing without pulmonary vascular imaging, which meant we selected a population with higher pretest probabilities. We included positive CUS because CUS is used to diagnose deep vein thrombosis (DVT) in pregnant patients with suspected PE, which allows a presumptive PE diagnosis and anticoagulation without requiring pulmonary vascular imaging in stable patients [[6], [7], [8],^10^].

### 2.3 Data collection

Seven abstractors blind to the study hypotheses undertook manual medical records review after completing structured training on data collection methods using a standardized computerized data collection tool, as in prior PE studies [25]. The principal investigator answered abstraction questions throughout the study. Abstractors identified unstructured variables (eg, PE-related symptoms), and our data analyst extracted structured variables (eg, demographics and vital signs) from KP’s administrative and clinical databases. Patient and clinical characteristics include demographics, comorbidities, PE- and DVT-related symptoms as documented by the treating clinician, vital signs, laboratory, imaging, and pharmacotherapy. Race and ethnicity were self-reported. We captured the most extreme vital signs in the direction of concern during the index diagnostic encounter.

Diagnostic settings included ED, L&D (an inpatient unit that also conducts triage and acute care), and outpatient clinics (obstetric, primary, and specialty care). Intervals were calculated from timestamps in the electronic health record. We defined imaging turnaround time from the order of initial VTE imaging (CUS or pulmonary vascular imaging) to imaging completion (either CUS positive for DVT or pulmonary vascular imaging). For patients undergoing serial imaging, eg, CUS followed by CTPA, the turnaround time was measured from the time of CUS order to the completion of CTPA. Imaging completion is a conservative end-point that identifies when results were accessible. Time from imaging completion to awareness by treating clinicians is not included, as it varied widely and was inconsistently reported.

A second reviewer, blind to the results of the index reviewer, undertook manual chart review of randomly selected cases (totaling 10% of the cohort) to evaluate interrater reliability using kappa values and percent agreement. We included the site of care (ED vs L&D vs clinic) and the following binary variables: reception of preemptive anticoagulation (the primary outcome) as well as 5 unstructured components of the pregnancy-adapted Geneva score: surgery under general anesthesia or lower limb fracture in the past 30 days; history of prior DVT or PE; unilateral lower limb pain; hemoptysis; and pain on lower limb palpation and unilateral edema on examination [29].

### 2.4 Outcomes

The primary outcome was receipt of preemptive anticoagulation prior to completion of definitive diagnostic imaging, either therapeutic-dose enoxaparin (approximately 1 mg/kg or higher) or intravenous (i.v.) unfractionated heparin. Administration of preemptive anticoagulation was identified independently by 2 complementary mechanisms: (1) by our analyst using clinical databases and (2) by abstractors during manual chart review. Any differences were adjudicated by the principal investigator. The secondary outcome was an estimation of eligibility for preemptive anticoagulation using criteria from the 2012 CHEST guidelines [15]. CHEST criteria are based on the estimated turnaround time of imaging and the degree of PE suspicion (high, intermediate, or low) (see Table 1 for CHEST recommendations) [29]. Magnitude of preimaging clinical suspicion is not routinely documented. As a proxy, we calculated pretest probabilities using the pregnancy-adapted Geneva score [29]. Studies in nonpregnant patients have used revised Geneva scores in a similar fashion [30,31]. The CHEST guidelines recommend “use of validated prediction models for probability of having…PE” to “usefully inform this assessment” of clinical suspicion [15]. We did not calculate objective risk of bleeding, which likely was low in this relatively young, healthy population. Among recipients of preemptive anticoagulation, we reviewed health records to identify hemorrhagic complications within 7 days.

**Table 1:** Eligibility for preemptive anticoagulation using recommendations from the 2012 guidelines of the American College of Chest Physicians (CHEST)^a^ [15].

	Risk-based indications for preemptive anticoagulation while awaiting definitive PE diagnostic imaging results in pregnancy (*N* = 326)		
	Low suspicion for PE (PAG score 0-1) *n* = 174	Intermediate suspicion for PE (PAG score 2-6) *n* = 140	High suspicion for PE (PAG score ≥ 7) *n* = 12
**Strata-specific CHEST indications for preemptive anticoagulation in patients at low risk for bleeding**	If results expected to be delayed >24 h	If results expected to be delayed >4 h	Delay duration irrelevant: anticoagulate all while awaiting results
Eligible for preemptive anticoagulation, *n* (%)	1 (0.6)	21 (15)	12 (100)
	Total eligibility based on summing the 3 risk strata above: 34 (10.4% of 326)		

PAG, Pregnancy-adapted Geneva [29]; PE, pulmonary embolism.

a The 2012 CHEST guidelines explain the 2 principles on which their recommendations are based. First, the higher the clinical suspicion for venous thromboembolism, the shorter the acceptable interval without treatment until results of diagnostic testing become available. Second, the higher the risk of bleeding, the longer the acceptable interval without treatment until results are available [15].

### 2.5 Statistical analysis

We presented continuous variables as medians with IQRs and categorical data as frequencies and proportions. Data management was conducted in SAS (version 9.4; SAS Institute, Inc, Cary, NC). All analyses were conducted in R version 4.3.1.

## 3 Results and Discussion

### 3.1 Study cohort

We identified 864 APED-eligible patients who underwent at least 1 of 4 VTE diagnostic tests during the study period (Figure). We excluded 144 patients with COVID-19 and an additional 394 patients who failed to undergo definitive diagnostic imaging. Patient characteristics and imaging turnaround times of the 326-patient study cohort are reported in Table 2. Median time from patient arrival at the ED or L&D to D-dimer order (when applicable) was 48.0 minutes (IQR, 33.0-80.0) and from patient arrival at the ED or L&D to initial VTE imaging order was 120.5 minutes (IQR, 72.5-185.8).

**Figure:**
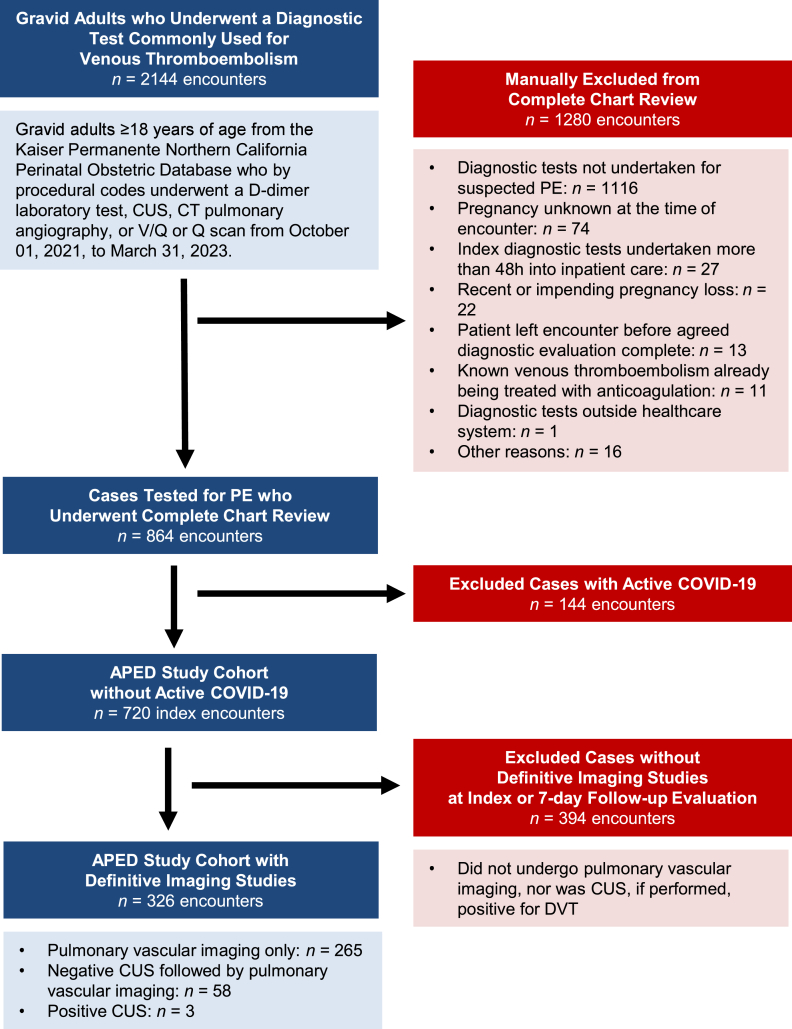
Cohort assembly. APED, Antenatal Pulmonary Embolism Diagnostics; CT, computed tomography; CUS, compression ultrasonography; DVT, deep vein thrombosis; PE, pulmonary embolism; V/Q, ventilation/perfusion.

**Table 2:** Patient and clinical characteristics of pregnant patients undergoing imaging studies for venous thromboembolism.

Patient and Clinical Characteristic	*N* = 326 *n* (%)^a^
**Age, median (IQR), y**	31.0 (26.5-35.5)
**Age category, y**	
<35	227 (70)
≥35	99 (30)
**Race and ethnicity, self-reported**	
Asian	45 (14)
Black or African American	44 (13)
Hispanic or Latino	97 (30)
Non-Hispanic White	82 (25)
Other race categories^b^	58 (18)
**Need for interpreter, self-reported**	7 (2)
**Health insurance**	
Commercial	256 (79)
Medicaid	70 (21)
**Socioeconomic status**^c^	
Not low	233 (71)
Low	81 (25)
Unknown	12 (4)
**Gravidity**	
1	76 (23)
≥2	250 (77)
**Parity**	
0	102 (31)
1	100 (31)
≥2	124 (38)
**Comorbidities**	
**Prepregnancy body mass index,** kg/m^2^	
<30	152 (47)
30-39	108 (33)
≥40	66 (20)
**Gestational diabetes**	17 (5)
**History of venous thromboembolism**	19 (5.8)
**Gestational age, trimester** (wk)	
First (<14)	41 (13)
Second (14-27)	118 (36)
Third (≥28)	167 (51)
**PE-related symptoms** (not exclusive)	
Shortness of breath	253 (78)
Thoracic pain	217 (67)
Palpitations	73 (22)
Syncope or presyncope	22 (7)
Hemoptysis	7 (2)
**DVT-related symptoms**	
Absent	311 (95)
Present	15 (5)
**Maximal heart rate,** beats/min	
<110	197 (60)
≥110	129 (40)
**Pulse oximetry, %**	
95-100	306 (94)
<95	16 (5)
Unknown	4 (1)
**Diagnostic setting**	
Emergency department	254 (78)
Labor and Delivery	65 (20)
Outpatient clinic	7 (2)
**Pretest probability (Pregnancy-adapted Geneva score)**^d^	
Low (0-1 points)	174 (53)
Intermediate (2-6 points)	140 (43)
High (≥7 points)	12 (4)
**D-dimer testing**	
Not obtained	117 (36)
Obtained	209 (64)
Values, mg/L	
<0.5	4 (1)
≥0.5 and <1.0	64 (20)
≥1.0	141 (43)
**Pulmonary vascular imaging study**	
CTPA alone	244 (75)
Lung scintigraphy alone	21 (6)
CUS alone (when positive for deep vein thrombosis)	3 (1)
Sequential negative CUS followed by pulmonary vascular imaging	58 (18)
**Turnaround times by imaging study and diagnostic setting,** median (IQR), h	
**Positive CUS**, *n* = 3	
Emergency department, *n* = 3	1.5 (1.0, 1.6)
Labor and Delivery, *n* = 0	N/A
Outpatient clinic, *n* = 0	N/A
**Negative CUS followed by pulmonary vascular imaging** (serial imaging), *n* = 58	
Emergency department, *n* = 47	3.5 (2.6, 5.0)
Labor and Delivery, *n* = 10	7.2 (3.5, 14.0)
Outpatient clinic, *n* = 1	23.7
**CTPA alone**, *n* = 244	
Emergency department, *n* = 189	1.4 (0.8, 2.0)
Labor & Delivery, *n* = 51	2.2 (1.3, 3.1)
Outpatient clinic, *n* = 4	13.7 (3.3, 41.7)
**Lung scintigraphy alone**, *n* = 21	
Emergency department, *n* = 15	3.4 (2.5, 6.4)
Labor & Delivery, *n* = 4	2.8 (1.6, 3.8)
Outpatient clinic, *n* = 2	21.1 (20.2, 22.0)
**Turnaround times of definitive PE imaging diagnostics, by setting,** h	
**Emergency department**	***n* = 254**
<1	66 (26)
≥1 and <4	155 (61)
≥4 and <24	33 (13)
≥24	0
**Labor and Delivery**	***n* = 65**
<1	5 (8)
≥1 and <4	46 (71)
≥4 and <24	13 (20)
≥24	1 (2)
**Outpatient clinic**	***n* = 7**
<1	0
≥1 and <4	2 (29)
≥4 and <24	4 (57)
≥24	1 (14)
**Turnaround times of definitive PE imaging diagnostics, by pretest probability**, cases stratified by times in h	
Low pretest probability, *n* = 174	
<1	41 (24)
≥1 and <4	105 (60)
≥4 and <24	27 (16)
**≥24**	1 (0.6)
Intermediate pretest probability, *n* = 140	
<1	28 (20)
≥1 and <4	91 (65)
**≥4 and <24**^e^	20 (14)
**≥24**^e^	1 (0.7)
High pretest probability, *n* = 12	
**<1**	2 (17)
**≥1 and <4**	7 (58)
**≥4 and <24**	3 (25)
**≥24**	0 (0)

Bold values indicate cases in which preemptive anticoagulation was indicated according to CHEST guidelines.

CHEST, American College of Chest Physicians; CTPA, computed tomography pulmonary angiography; CUS, compression ultrasonography; DVT, deep vein thrombosis; PE, pulmonary embolism.

a *n* (%) throughout, except age and turnaround times, which are reported as median (IQR).

b Other race categories include American Indian/Alaskan Native (*n* = 3), Native Hawaiian or other Pacific Islander (*n* = 4) and decline to state (*n* = 9).

c We defined socioeconomic status as low by census block group (groupings of approximately 1000 residents) with 25% or more of adult residents having less than a high school education or 20% or more of households having an annual income below the federal poverty level.

d The Pregnancy-adapted Geneva score comprises 7 scored items that when summed reach a point score from 0 to 20: Age ≥40 years (1 point); surgery (under general anesthesia) or lower limb fracture in past month (2 points); previous DVT or PE (3 points); unilateral lower limb pain (3 points); hemoptysis (2 points): pain on lower limb palpation and unilateral edema (4 points); heart rate >110 bpm (5 points) [29].

e Among the 21 intermediate-risk cases with turnaround time ≥4 h who would have been eligible for preemptive anticoagulation, 12 were seen in the emergency department (3 with CT only, 3 with lung scintigraphy, 6 with serial imaging) and 9 were seen in Labor & Delivery (6 with CT only, 1 with lung scintigraphy, 2 with serial imaging).

Prevalence of presumed or confirmed PE was 2.5% (8/326): 3 were diagnosed by CUS with acute DVT and presumed PE and 5 patients had confirmed PE by CTPA. We report their times of imaging and anticoagulation in Table 3 to illustrate 2 intervals of delay that could have been obviated by preemptive anticoagulation had it been indicated (as it would under different society guidelines [Table 4]): (a) delay of imaging turnaround time and (b) delay to postimaging anticoagulation administration.

**Table 3:** Pretest probability and timing of imaging and anticoagulation in ambulatory pregnant patients diagnosed with acute venous thromboembolism.

Case	Pregnancy-adapted Geneva score pretest probability	Imaging	Imaging turnaround time^a^	Time to initial anticoagulation	
				From final imaging result	From initial imaging order
A	High	CUS alone	30 min	1 h 16 min	1 h 46 min
B	Intermediate	CUS alone	1 h 29 min	>1 h 49 min^b^	>3 h 18 min^b^
C	High	CUS alone	1 h 44 min	2 h 7 min	3 h 51 min
D	Low	CTPA alone	19 min	27 min	46 min
E	Intermediate	CTPA alone	1 h 4 min	3 min	1 h 7 min
F	Intermediate	CTPA alone	1 h 22 min	52 min	2 h 14 min
G	Intermediate	CTPA alone	3 h 1 min	3 h 59 min	7 h
H	Intermediate	CUS, then CTPA	5 h 32 min	3 h 2 min	8 h 34 min

CTPA, computed tomography pulmonary angiography; CUS, compression ultrasonography.

a Turnaround time measured from order of initial imaging study to result of final imaging study.

b This patient was discharged without having received an initial dose of anticoagulation. Time to initial anticoagulation in this case is unknown but was beyond discharge, which occurred 1 h 49 min after final imaging result.

**Table 4:** Professional society recommendations on preemptive anticoagulation during pulmonary embolism diagnostics.^a^

Society, short title (country or region, year of publication)	Recommendations	Strength of recommendation and level of evidence^b^	Assumptions and eligibility: n (%)
**For General Patients** (limited to the United States)			
American College of Chest Physicians, Antithrombotic Therapy for VTE Disease (United States, 2012^b^) [15]	Recommendations stratified by clinical suspicion: ● High: we suggest treatment with parenteral anticoagulants compared with no treatment while awaiting the results of diagnostic tests ● Intermediate: we suggest treatment with parenteral anticoagulants compared with no treatment if the results of diagnostic tests are expected to be delayed for more than 4 h ● Low: we suggest not treating with parenteral anticoagulants while awaiting the results of diagnostic tests, provided test results are expected within 24 h.	Recommendation: weak Level of evidence: low quality.	No assumptions necessary Eligibility: 34 (10.4) (see Table 1 above)
American Heart Association, Management of Massive and Submassive PE (United States, 2011) [17]	● Therapeutic anticoagulation during the diagnostic workup should be given to patients with intermediate or high clinical probability of PE and no contraindications to anticoagulation.	Recommendation: Treatment should be administered; benefit >>>risk) Level of evidence: only expert opinion, case studies, or standard of care.	No assumptions necessary Eligibility: 152 (46.6)
**For Pregnant Patients** (selected from around the world)			
Society of Obstetric Medicine of Australia and New Zealand, PE in Pregnancy and Post-Partum (Australia and New Zealand, 2021) [9]	● Therapeutic LMWH should only be administered prior to pulmonary imaging in hemodynamically unstable women or if pulmonary imaging is not immediately available.	Consensus-based recommendation: Where there was insufficient evidence, the expert development group made clinical consensus recommendations.	Assumption: “not immediately available” means results delayed ≥1 h Eligibility: 304 (93.3)
2018 ESC Guidelines for the management of cardiovascular diseases during pregnancy (Europe, 2018) [10] See also Figure 7 in 2019 ESC Guidelines for the diagnosis and management of acute pulmonary embolism developed in collaboration with the European Respiratory Society (ERS) (Europe, 2019) [11]	● A high index of suspicion is important, and all pregnant women with signs and symptoms suggestive of VTE^c^ should have objective testing performed urgently and receive therapeutic anticoagulation until the diagnosis is established.	Strength of recommendation and level of evidence not specified.	No assumptions necessary Eligibility: 326 (100)
Working Group in Women’s Health of the Society of Thrombosis and Haemostasis, Diagnosis of pregnancy-associated VTE (Germany, 2016) [13]	● If there is a delay in performing objective testing, anticoagulant therapy should be initiated until VTE is objectively confirmed or excluded, unless there are strong contraindications against the use of anticoagulants. ● A clinical suspicion of pregnancy-associated VTE requires immediate objective testing to confirm or exclude DVT and/or PE. If the probability of VTE is estimated to be high or if there is any delay in obtaining test results, therapeutic doses of LMWH or unfractionated heparin. should be administered until a definitive decision based on objective test results can be made. ● Their algorithm opens as follows: “Consider to start [AC] during diagnostic work-up.”	Strength of recommendation and level of evidence not specified.	Assumption: “a delay” means results delayed ≥1 h Eligibility: all high pretest probability patients plus low-to-intermediate pretest probability patients with delay ≥1 h: 257 (78.8)
Royal College of Obstetricians and Gynaecologists, Thrombosis and Embolism during Pregnancy and the Puerperium (United Kingdom, 2015) [14]	● Any woman with symptoms and/or signs suggestive of VTE should have objective testing performed expeditiously and treatment with LMWH given until the diagnosis is excluded by objective testing, unless treatment is strongly contraindicated. ● In clinically suspected VTE, LMWH should be postponed until objective testing has confirmed the diagnosis in women at risk of bleeding after careful consideration of the balance of risks of hemorrhage and clotting.	Strength of recommendation and level of evidence for preemptive anticoagulation not specified.	No assumptions necessary Eligibility: 326 (100)
American Thoracic Society and Society of Thoracic Radiology, Evaluation of suspected PE in pregnancy (United States, 2011) [16]	● For a patient in whom there is a high clinical suspicion of PE and a low risk of bleeding, anticoagulant therapy is recommended while awaiting the outcome of diagnostic tests (strong recommendation, low-quality evidence) ● In real-life situations where either the patient is unstable or some studies are not available on a timely basis, empiric initiation of therapy and/or alternate diagnostic strategies should be considered.	Recommendation: strong Level of evidence: low quality.	No assumptions necessary for high-suspicion patients Assumption: “not available on a timely basis” means results delayed ≥4 h Eligibility: 59 (18.0)

LMWH, low molecular weight heparin; PE, pulmonary embolism; VTE, venous thromboembolism.

a This table is not exhaustive; we included select examples of recent (2011–2024) English-language guidelines to illustrate variation.

b Specification of the strength of the recommendation and the level of evidence was inconsistently reported in the guidelines. When specified, the level of evidence was uniformly acknowledged to be low quality, and the strength of the recommendation varied from weak to strong. Simply providing a recommendation for preemptive anticoagulation implies that the society thought benefits outweighed risks for the population in question.

c Suspected PE during pregnancy, defined as “High pretest probability or low-to-intermediate probability and positive D-dimer result.”

### 3.2 Outcomes

Two patients (0.6%) received preemptive anticoagulation, both identified by our analyst and our abstractors. The first was a 20-year-old at 22 weeks who presented to the ED at night with PE symptoms, normal vital signs, and low pretest probability for PE. The physician ordered Q scintigraphy, which would be delayed at least 6 to 8 hours, and 120 mg of subcutaneous enoxaparin. Turnaround time from imaging order to its completion was 9.5 hours. The second patient was a 33-year-old with known asthma at 20 weeks who presented to L&D in moderate respiratory distress with pulse oximetry of 90% (normal ≥92%) and wheezing on chest auscultation. Her dyspnea rapidly improved with oxygen administration; she was also treated with bronchodilators. The physician ordered CTPA and i.v. unfractionated heparin but did not document their magnitude of suspicion for a PE diagnosis. The patient’s pretest probability for PE was calculated to be intermediate. CTPA turnaround time was 98 minutes. Neither patient was diagnosed with PE nor experienced a hemorrhagic complication.

Overall, we identified 34 patients (10.4%) who were retrospectively deemed eligible for preemptive anticoagulation using CHEST criteria (Table 1). The 2 patients described above who had received preemptive anticoagulation did not meet formal CHEST criteria. One of the 34 eligible patients was diagnosed with PE.

We were not surprised to find that outpatient clinics had more prolonged imaging turnaround times than hospital-based departments (ED and L&D) (Table 2). These setting-specific differences in imaging delays could have a bearing on the application of preemptive anticoagulation, which is often based on expected turnaround times.

Thirty-three cases (10% of the study cohort) underwent a second abstraction for 8 variables. All variables had perfect agreement (Cohen’s kappa for 2 raters = 1). Interrater reliability results were similar to those from a prior APED study [22].

### 3.3 Studies of preemptive anticoagulation in nongravid patients

Though no studies have examined preemptive anticoagulation in pregnant patients, several have examined this treatment in nonpregnant patients [[30], [31], [32]]. They found low prevalence of use. The largest was a 12-center prospective U.S. study of 7932 ED adults who, from July 2003 to November 2006, underwent PE testing, including isolated D-dimer testing [32]. Overall, 342 (4.3%) patients received preemptive anticoagulation. PE was diagnosed in 481 (6.1%) patients. Hemorrhagic complications were low (<1%), and no more prevalent among those treated than not treated with preemptive anticoagulation. The second study included 392 ED and hospital patients in Australia who underwent CTPA from April through June of 2019 [31]. Among 144 ED patients, 4 (2.8%) received preemptive anticoagulation and 4 (2.8%) were diagnosed with PE.

### 3.4 Eligibility varies widely across society guidelines

Eligibility for preemptive anticoagulation varies widely across society guidelines (Table 4) [9,10,12,13,16,17]. Complicating eligibility determination are nonspecific temporal thresholds. Some guidelines recommend preemptive anticoagulation when definitive imaging is “not immediately available” or not available “in a timely fashion” (Table 4). To estimate eligibility, we defined nonspecific time frames, as noted in Table 4. Based on our interpretative assumptions, eligibility in our study population ranged from 10.4% to 100%, depending on the guideline in question.

In addition to the varying criteria crafted by society experts, we identified 2 modeling studies used to calculate criteria for preemptive anticoagulation among nonpregnant patients [33,34]. In the literature, we did not identify modeling using risk/benefit assumptions geared specifically to pregnant patients.

### 3.6 Next steps

On the research front, studies are needed to understand preemptive anticoagulation use among pregnant patients in other settings, settings that differ from our own in PE prevalence and accessibility to diagnostic imaging, as well as settings whose prevailing guideline recommendations differ from CHEST (Table 4). Investigators should explore the risks and benefits of preemptive anticoagulation in pregnant patients undergoing PE diagnostics. Also worth exploring are variations in time intervals from PE diagnosis to initiation of treatment, which we described in our small cohort (Table 3).

Until higher quality evidence is forthcoming, clinicians, departments, hospitals, and health systems may benefit from examining the literature, their setting’s features, population’s characteristics, and practice patterns to determine which approach to preemptive anticoagulation seems best suited to their circumstances.

### 3.7 Limitations

The study had several limitations. First, our population is not comprehensive: we did not include patients suspected of PE who failed to undergo definitive VTE imaging studies. We cannot address the prevalence of preemptive anticoagulation among patients suspected of PE who received only D-dimer testing or no testing whatsoever. However, in a separate APED study of 91 patients who declined recommended pulmonary vascular imaging, none were administered preemptive anticoagulation [22]. Second, the magnitude of PE suspicion held by the diagnosticians was rarely documented; we used post hoc calculations of pretest probability scores as a proxy, as others have done [30,31]. Third, some patients may have had contraindications to anticoagulation. These were not included in this analysis. Many bleeding risks, however, are uncommon in this relatively young and healthy population [35]. For example, in a separate cohort of over 200,000 pregnant persons in KP Northern California, only 0.1% had chronic renal failure [36]. Thrombocytopenia <80,000 per cubic mm also is rare in pregnancy (0.1%) and a history of heparin-induced thrombocytopenia even more so [37,38]. Fourth, by not including in our calculation of overall turnaround times preimaging D-dimer testing, we may have underestimated the proportion of intermediate-risk patients eligible for anticoagulation by the CHEST recommendations. Fifth, we used *actual* delays in our estimates of eligibility, whereas in practice, clinicians would use *expected* delays. Lastly, given the influence of local factors on preemptive anticoagulation use and eligibility, our results may not be generalizable to other settings with different patients, imaging and anticoagulation practices, and relevant society guidelines.

## 4 Conclusions

In this retrospective cohort study across 21 U.S. community medical centers, we found rare use (<1%) of preemptive anticoagulation among patients undergoing antenatal PE diagnostic imaging. These results may have been anticipated, considering the low-quality evidence and diversity of expert opinions found in society guidelines. Rare use of preemptive anticoagulation is also explicable in a population with low PE prevalence and ready access to diagnostic imaging. More research is needed to explore setting-specific variations in the use of preemptive anticoagulation, to undertake modeling studies with assumptions particular to pregnancy, and to provide evidence of the risks and benefits of preemptive anticoagulation in pregnancy.
