# Corrigendum to ‘Preemptive anticoagulation during antenatal pulmonary embolism diagnostics in a community setting: retrospective cohort study’ [Research and Practice in Thrombosis and Haemostasis Volume 9, Issue 1, January 2025, 102695]

**DOI:** 10.1016/j.rpth.2025.102892

**Published:** 2025-05-30

**Authors:** Aidan R. Campbell, Cole J. Florio, Grace V. Heringer, Sara T. Woldemariam, Scott D. Casey, William B. Stubblefield, Lauren M. Westafer, Edward Qiao, Cydney E. Middleton, Lara Zekar, Nachiketa Gupta, Madeline J. Somers, Mary E. Reed, Nareg H. Roubinian, Ashok P. Pai, Jeffrey D. Sperling, David R. Vinson

**Affiliations:** 1 Kaiser Permanente CREST Network, Pleasanton, California, USA; 2 Department of Microbiology and Molecular Genetics, University of California, Davis, California, USA; 3 Department of Neurobiology, Physiology and Behavior, University of California, Davis, California, USA; 4 Department of Obstetrics and Gynecology, Kaiser Permanente Oakland Medical Center, Oakland, California, USA; 5 Kaiser Permanente Northern California Division of Research, Pleasanton, California, USA; 6 Department of Emergency Medicine, Kaiser Permanente Vallejo Medical Center, Vallejo, California, USA; 7 The Permanente Medical Group, Pleasanton, California, USA; 8 Department of Emergency Medicine, Vanderbilt University Medical Center, Nashville, Tennessee, USA; 9 Department of Emergency Medicine and Department of Healthcare Delivery and Population Science, University of Massachusetts Chan Medical School-Baystate, Springfield, Massachusetts, USA; 10 California Northstate University College of Medicine, Elk Grove, California, USA; 11 Department of Emergency Medicine, UC Davis Health, Sacramento, California, USA; 12 Department of Emergency Medicine, Kaiser Permanente Redwood City Medical Center, Redwood City, California, USA; 13 Department of Pulmonary and Critical Care Medicine, Kaiser Permanente Oakland Medical Center, Oakland, California, USA; 14 Department of Hematology and Oncology, Kaiser Permanente Oakland Medical Center, Oakland, California, USA; 15 Department of Maternal and Fetal Medicine, Kaiser Permanente Modesto Medical Center, Modesto, California, USA; 16 Department of Emergency Medicine, Kaiser Permanente Roseville Medical Center, Roseville, California, USA

The authors regret miscategorization of race/ethnicity in Table 2. These corrections do not alter the results or conclusions of the study.

**Table:** 

Race and ethnicity, self-reported	
Asian	53 (16)
Black or African American	51 (16)
Hispanic or Latino	116 (36)
Non-Hispanic White	95 (29)
Other race categories^b^	11 (3)

^b^ Other race categories include American Indian/Alaskan Native (n=3), Native Hawaiian or Other Pacific Islander (n=2), decline to state (n=6)

The authors would like to apologise for any inconvenience caused.

